# Gender-Based Quantitative Analysis of the Grand Piano Sign in Mechanically Aligned Total Knee Arthroplasty in Asians

**DOI:** 10.3390/jcm10091969

**Published:** 2021-05-04

**Authors:** Byung-Woo Cho, Ji-Hoon Nam, Yong-Gon Koh, Ji-Hwan Min, Kwan-Kyu Park, Kyoung-Tak Kang

**Affiliations:** 1 Department of Orthopedic Surgery, Severance Hospital, Yonsei University College of Medicine, 50-1 Yonsei-ro, Seodaemun-gu, Seoul 03722, Korea; chobw0704@yuhs.ac (B.-W.C.); MINJH8811@yuhs.ac (J.-H.M.); 2 Department of Mechanical Engineering, Yonsei University, 50 Yonsei-ro, Seodaemun-gu, Seoul 03722, Korea; namjh8901@naver.com; 3 Joint Reconstruction Center, Department of Orthopaedic Surgery, Yonsei Sarang Hospital, 10 Hyoryeong-ro, Seocho-gu, Seoul 06698, Korea; osygkoh@gmail.com

**Keywords:** Korean patients, grand piano sign, morphometry, total knee replacement

## Abstract

In mechanically aligned (MA) total knee arthroplasty (TKA), the grand piano sign helps surgeons to further ensure the proper external rotation of the femoral component. The goal of this study was to determine the sex-related differences in the shape of the anterior resection surface using 3D magnetic resonance imaging (MRI) models. MRI scans were performed on 267 consecutive patients (202 women and 65 men) with osteoarthritis who underwent TKA in order to reconstruct a 3D model. Virtual anterior condylar resection was performed based on the surgical transepicondylar axis (sTEA), Whiteside’s line (WSL), and flexion-extension axis (FEA). On the anterior resection surface, both lateral length (LatL) and medial length (MedL) were measured, and the ratio between the two (MedL/LatL) was calculated. The mediolateral width of the distal femur (ML) and anterior resection surface (M′L′) were measured, and the ratio between the M′L′ and ML (M′L′/ML) was calculated. Both the lateral deviation (LD) and the ratio between LD and ML (LD/ML) were also determined. Morphological classification of the anterior resection surface was conducted based on the presence of a definite medial peak. When based on the sTEA or WSL, the MedL/LatL of female subjects was significantly greater than that of male subjects (*p* < 0.001 and *p* < 0.05, respectively). The MedL/LatL of the FEA was consistently larger than that obtained using the sTEA or WSL. Among female subjects, the MedL/LatL of the sTEA was significantly greater than that of the WSL, although this was not the case in either the total study population or the male subjects alone. When based on the sTEA, the M′L′/ML was statistically greater in the female subjects (*p* < 0.01). The LD was greater in the male subjects (*p* < 0.01), but there was no difference between the male and female subjects when comparing the LD/ML (*p* = 0.93). The proportion of double- and single-peak types was not significantly different between the sexes (*p* = 0.196). Surgeons should be aware that the shape of the anterior resection surface may differ depending on the sex of the patient. The results of this study provide more consistent surgical outcomes as well as fundamental anatomical data for designing suitable prostheses applicable to the Korean population.

## 1. Introduction

### 1.1. Background

Proper rotational alignment of the femoral component during mechanically aligned (MA) total knee arthroplasty (TKA) is essential for successful outcomes. Malrotation of the femoral component has been shown to induce instability and stiffness due to flexion gap asymmetry [1,2], as well as patellar maltracking, anterior notching, and pain [3,4,5,6]. When using the measured resection technique, the degree of femoral rotation is determined based on bony landmarks, such as the transepicondylar axis (TEA), posterior condylar axis (PCA), Whiteside’s line (WSL), and flexion-extension axis (FEA). Among these landmarks, the use of TEA consistently recreated a balanced flexion gap and it has been considered to be in close proximity to the optimal flexion axis, regardless of lower extremity alignment [7,8,9]. Despite these findings, it is difficult to accurately locate the TEA during actual surgery. In a cadaveric study, Stoeckl et al. reported that there were large differences in the location of the epicondyle as designated by four experienced orthopedic surgeons [10]. Wai et al. reported a significant difference between the TEA, as determined in dissected cadavers, and those found using computed tomography (CT) images [11]. Additionally, it can be difficult to find bony landmarks due to the deformity caused by severe osteoarthritis (OA) [12]. For this reason, the instruments used in real surgery are often based on the PCA. However, the PCA is also not an accurate reference as its angular difference in relation to the TEA differs according to ethnicity and sex; moreover, hypoplasia of the lateral condyle is often present in patients with OA. As a result, it can be difficult to determine the degree of rotation with only one point of reference. Thus, surgeons can use the grand piano sign to further ensure the proper degree of external rotation of the femoral component by assessing the shape of the anterior resection surface. However, since the judgment of the proper shape of the anterior resection surface is inherently subjective, it may differ from one surgeon to another. In an attempt to address this issue, Cui et al. established an objective benchmark by quantitatively evaluating the shape of the anterior resection surface using three-dimensional (3D) CT simulation [13]. However, their study did not consider sex differences in the morphology of the anterior resection surface.

### 1.2. Rationale

The anatomical proportions of the human knee have been shown to vary according to sex [14,15,16,17,18,19,20,21,22]. This finding serves as the basis for the development of implants that cater to the morphology of each sex. Therefore, it is conceivable that the shape of the anterior resection surface should reflect this tendency. The goal of this study was to determine the sex-related differences in the shape of the anterior resection surface using 3D magnetic resonance imaging (MRI) models. We hypothesized that the quantitative values of the anterior resection plane differ according to sex.

## 2. Materials and Methods

Institutional review board approval was obtained for this study. After excluding patients with any history of infection, fracture, osteotomy, or rheumatoid arthritis, we performed MRIs on a total of 267 consecutive patients (202 women and 65 men) with OA who were undergoing TKA at our institution. Subject characteristics, such as age, body mass index (BMI, kg/m^2^), and sex differences in lower extremity alignment, were recorded (Table 1).

We screened end-stage OA patients awaiting TKA using a standard protocol and a 1.5 T MRI scanner (Achieva 1.5 T; Philips Healthcare, Best, The Netherlands). The images were obtained using a 1 mm high-resolution slice thickness in the sagittal plane of the tibiofemoral knee joint, whereas images of the hip and ankle joints were taken with a 5 mm slice thickness in the axial plane. This MRI technique, used in patient-specific instruments, allowed us to effectively reconstruct a 3D model [23].

The 3D femur model was created using a superimposed MRI segment as a mask. Segmentation and reconstruction were conducted using K-RECON (AIES, Uijeoungbu, KOREA). The 3D femur model was imported into the surgical planning software K-PLAN (AIES, Uijeoungbu, KOREA). Surgical planning of MA TKA was conducted using the measured resection technique [24]. Surgical simulation was performed using a prosthesis from Legion (Smith and Nephew, Memphis, TN, USA). Distal femoral cutting was performed perpendicular to the mechanical axis of the femur, and the knee center was set as an intramedullary rod entry [25]. The flexion angle of the anterior flange was set to 3° [26], and the distal and posterior femoral resections were 9 mm in size. The surgical transepicondylar axis (sTEA), WSL, and FEA were used as references for femoral rotation based on previous reports [27,28,29]. The sTEA was defined as the line between the most prominent point on the lateral femoral epicondyle and the sulcus of the medial femoral epicondyle. The WSL was defined as a line connecting the deepest point on the trochlear groove and the highest point of the intercondylar notch. The FEA was defined as a line connecting the center of the medial and lateral best-fitted circles in the sagittal plane. Virtual anterior condylar resection was performed based on these points of reference.

**Figure 1:**
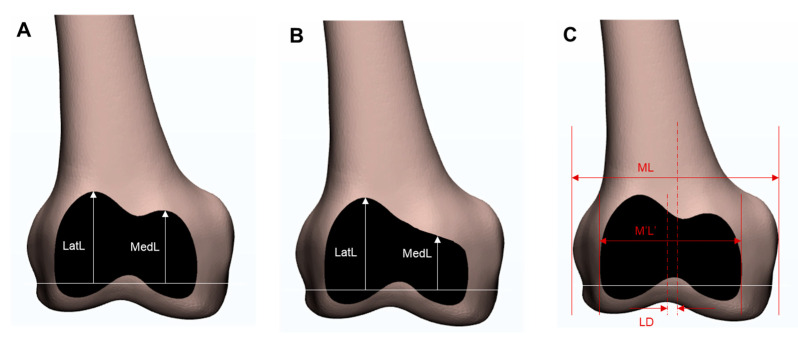
The measurements of the anterior resection surface. (**A**) The ratio between the lateral and medial lengths (MedL/LatL). (**B**) If there was no definite medial peak, the point of inflection was used instead. (**C**) The mediolateral widths of the distal femur (ML) and anterior resection surface (M′L′). The distance between the midpoints of the ML and M′L′ was defined as the lateral deviation (LD).

On the anterior resection surface, both the lateral length (LatL) and medial length (MedL) were measured. The length was defined as the distance between the distal cutting line and either the lateral or medial peak point (Figure 1A), and the ratio between the lateral and medial lengths (MedL/LatL) was calculated. In the absence of a medial peak, the point of inflection was used instead (Figure 1B). The mediolateral width of both the distal femur (ML) and anterior resection surface (M′L′) was measured (Figure 1C), and the ratio between the M′L′ and ML (M′L′/ML) was calculated. The lateral deviation (LD) was defined as the distance between the midlines of the ML and M′L′ (Figure 1C). The ratio between the LD and ML (LD/ML) was then calculated. The morphology of the anterior resection surface was classified based on the presence of a definite medial peak. Patients were classified as either having a double peak (Figure 1A) or single peak (Figure 1B). To assess intra- and interobserver variability, 100 3D MRI scans from 50 female and 50 male patients were re-measured more than 1 month after the initial measurement by the same observer and by a second observer. The intraobserver error (0.87) and interobserver error (0.91) were calculated using the intraclass correlation method.

Statistical analysis was conducted using the R statistical software (version 3.6.3). We used both Student′s *t*-test and the chi-square test to compare the ratios between sexes, and an analysis of variance was used to compare the ratios between the three bony references. To counteract the problem of multiple comparisons, the Bonferroni correction was used. Significant differences were defined as a *p*-value of less than 0.05. Power analysis was performed using G power 3.1 software. Group parameters were set as the ratios between the lateral and medial heights of both men and women. The calculated statistical power was 100%, and the alpha value of 0.05 was used as the input parameter. The statistical power of previous studies was reported to be 80% [30,31].

## 3. Results

Table 2 shows the difference in MedL/LatL between sexes according to the rotational references. When based on the sTEA and WSL, the MedL/LatL was significantly greater in the female subjects than in the male subjects (*p* < 0.001 and *p* < 0.05, respectively). There was no statistical difference between the sexes when the MedL/LatL was based on the FEA (*p* = 0.86).

Table 3 shows the MedL/LatL data compared between the rotational references. In all cases, the MedL/LatL was larger when FEA was used as a reference than when sTEA or WSL was used. In the entire study population and the male subjects alone, no difference was observed between the MedL/LatL when using either sTEA or WSL; however, in the female subjects, the MedL/LatL was significantly greater when using sTEA than when using the WSL.

Table 4 shows other comparisons between sexes based on sTEA. Both the M′L′ and ML were greater in male subjects than in female subjects (both *p* < 0.001); however, the M′L′/ML was statistically greater in the female subjects (*p* < 0.01). The LD was greater in the male subjects (*p* < 0.01), but there was no difference between sexes when comparing the LD/ML (*p* = 0.93). When classifying the shape of the anterior resection surface, the proportion of double peaks and single peaks did not show a statistical difference between sexes (*p* = 0.196).

## 4. Discussion

In our study using the 3D MRI model, we confirmed that the quantitative values of the anterior resection surface differs between sexes in Korean subjects. The MedL/LatL was greater in female subjects than in male subjects when using either the sTEA or WSL as a reference. In female subjects, the MedL/LatL was significantly greater when using the sTEA than when using the WSL; however, there was no difference between the entire study population or male subjects alone in this regard. When based on the sTEA, the M′L′/ML was statistically greater in female subjects.

Notably, the results may vary depending on the simulation method. The first quantitative analysis of the anterior resection surface was performed by Cui et al., who performed anterior resection without femoral distal resection [13]. Kim et al. [26] analyzed the anterior resection surface after performing distal femoral resection, similar to that in our study. Therefore, the ratio might have been underestimated compared with that of Cui et al.’s study.

**Figure 2:**
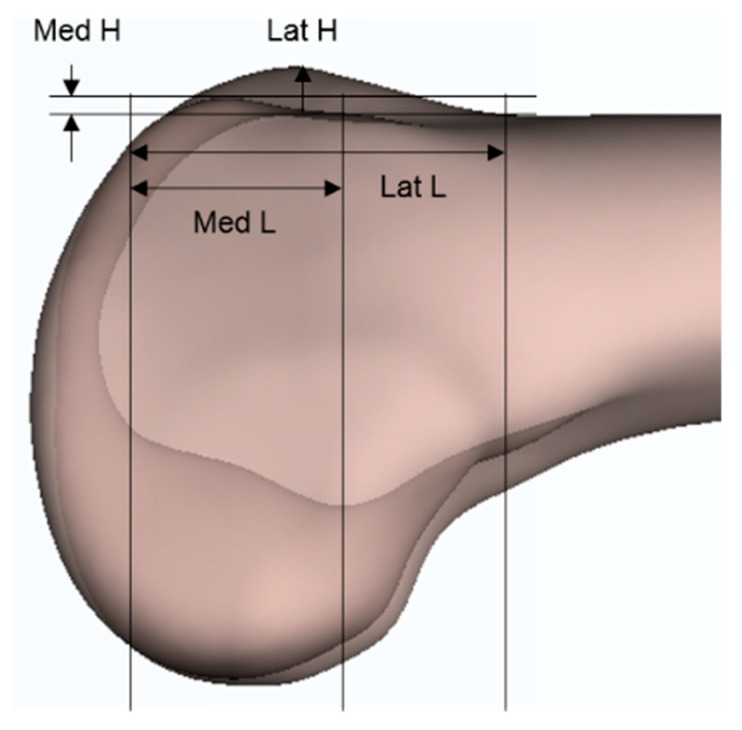
The lengths and heights of resected anterior condyles. The lateral aspect of resected anterior condyle was in the shape of a segment of a circle.

In our study, the MedL/LatL ratio of female subjects was greater than that of male subjects when anterior condylar resection was performed using sTEA or WSL as a reference. Since there were no previous studies about the length of the anterior resection surface, we assumed that the height and length are proportional because the lateral aspect of resected anterior condyle was in the shape of a segment of a circle (Figure 2). Table 5 shows the average values of the heights of anterior condyles according to sex in the literature. Studies by Koh et al. in the Korean population and by Yang et al. in the Chinese population both showed that the ratio was higher in women than in men [19,22]. However, the opposite result was found in a study conducted on the Western population [14,32]. Although there were differences in the data due to the selection of other rotational references, it is thought that the sex differences in the ratio may have been influenced by the ethnicities of the patients. Therefore, if our method of simulation were to be applied in studies conducted on Western populations, it is thought that the MedL/LatL will be larger in male subjects.

In Cui et al.’s study, quantitative analysis of the anterior resection surface revealed no difference between the MedL/LatL obtained using either sTEA or an external rotation of 3° in relation to the PCA; however, there was a statistical difference between the PCA and an external rotation of 6° in relation to the PCA [13]. In our study, the MedL/LatL did not statistically differ between the sTEA and WSL in the entire study population and male subjects alone; nonetheless, there was a difference between the sTEA and WSL in female subjects. Jang et al. reported an average difference of 1.9° between the WSL and sTEA, regardless of ethnicity or sex [34], and Reddy et al. reported that there was no statistical difference between Indian men and women when using either the WSL or sTEA [35]. Koh et al. reported no statistical difference in the groove angle of the proximal trochlea between men and women in a Korean study [22]. Taken together, all of these findings suggest that the sex differences in the MedL/LatL may be due to variations in the geometry of the anterior condyles rather than the angles of the WSL and sTEA. In addition, since the angle of the FEA statistically differed from those of the sTEA and WSL in the literature [34], a clear difference can be seen in the MedL/LatL.

In our study, the mediolateral widths (ML and M′L′) were greater in male subjects than in female subjects; however, the M′L′/ML was larger in the latter. These differences stem from the anatomical differences between sexes. In a study of Western populations, the size of the distal femur was smaller in women than in men, and the mediolateral width was also found to be narrower in the former [14,16]. The same pattern was observed in a study on Chinese patients [15,19] and another on Korean patients [18]. The shape of the distal femur in the axial cut also tended to be more trapezoidal in women than in men [14], and the ML was narrower at the same anterior-posterior length in the former. Hence, when anterior condyle resection was performed using this method, the M′L′/ML increased in women. However, the degree of lateral deviation of the anterior resection surface was not statistically different between sexes when the size of the distal femur was adjusted for.

Cui et al. reported that the surgeon can ensure a correct rotational alignment of the femoral component by using the quantified sign patterns of anterior resection surface (grand piano sign, boot sign, and butterfly sign) [13]. In our study, however, even if the anterior condylar resections were identically performed based on sTEA, the double-peak type (grand piano sign and butterfly sign) and single-peak type (boot sign) were mixed, and the proportion amongst the sexes did not show a statistical difference. Therefore, it is not recommended to confirm the rotation only by the shape of the anterior resection surface.

This study was subject to certain limitations. First, since our study only used Korean patients, the findings cannot be generalized to other ethnicities. Future studies are needed to confirm the findings in various populations. Second, the results may vary slightly depending on the instruments used. For example, a difference in the ratio may occur depending on differences in the anterior flange angle. Third, we did not assess postoperative clinical outcomes, as we did not study patients who had undergone TKA. Nevertheless, this study is, to the best of our knowledge, the first to report sex differences in the grand piano sign following anterior condylar resection in Asian MA TKA patients.

## 5. Conclusions

In our simulation study using a 3D MRI model, we confirmed that the shape of the anterior resection surface differed according to sex, a finding that surgeons should be made acutely aware of prior to operation. The results of this study provide more consistent surgical outcomes to surgeons, as well as contribute pertinent anatomical data for designing suitable prostheses applicable to the Korean population.

## Figures and Tables

**Table 1:** Comparison of age and BMI according to patient sex.

Parameter	All Patients (*n* = 267)	Women (*n* = 202)	Men (*n* = 65)	*p*-Value
	Mean ± SD (range)	Mean ± SD (range)	Mean ± SD (range)	
Age	70.9 ± 6.5 (54, 85)	71.3 ± 6.6 (58, 85)	69.9 ± 6.1 (58, 85)	n.s
BMI	23.2 ± 3.9 (15.9, 39.7)	23.0 ± 4.1 (16.6, 39.7)	23.5 ± 3.5 (15.9, 33.2)	n.s
Varus/Valgus	251/16	190/12	61/4	NA

BMI, body mass index; SD, standard deviation; n.s, not significant; NA, not applicable.

**Table 2:** MedL/LatL according to patient sex.

Parameter	All Patients (*n* = 267)	Women (*n* = 202)	Men (*n* = 65)	*p*-Value
	Mean ± SD	Mean ± SD	Mean ± SD	
sTEA	0.63 ± 0.11	0.65 ± 0.11	0.58 ± 0.08	<0.001
WSL	0.60 ± 0.11	0.61 ± 0.12	0.58 ± 0.09	<0.05
FEA	0.70 ± 0.14	0.70 ± 0.14	0.70 ± 0.14	0.86

MedL/LatL; ratio between the lateral and medial lengths; SD, standard deviation; sTEA, surgical transepicondylar axis; WSL, Whiteside line; FEA, flexion-extension axis.

**Table 3:** Sex differences in MedL/LatL according to the rotational references.

	Rotational References			*p*-Value (ANOVA)	Comparison
	sTEA	WSL	FEA		
All subjects	0.63 ± 0.11	0.60 ± 0.11	0.70 ± 0.14	<0.001	FEA > sTEA = WSL
Female	0.65 ± 0.10	0.61 ± 0.12	0.70 ± 0.14	<0.001	FEA > sTEA > WSL
Male	0.58 ± 0.08	0.58 ± 0.09	0.70 ± 0.14	<0.001	FEA > sTEA = WSL

MedL/LatL; ratio between the lateral and medial lengths; sTEA, Surgical transepicondylar axis; WSL, Whiteside line; FEA, flexion-extension axis; ANOVA, analysis of variance.

**Table 4:** Sex differences in the ML and M′L′ and their ratios (M′L′/ML), LD, LD/ML, and type of anterior resection surface shape based on sTEA.

Parameter	All Patients (*n* = 267)	Women (*n* = 202)	Men (*n* = 65)	*p*-Value
	Mean ± SD (range)	Mean ± SD (range)	Mean ± SD (range)	
ML (mm)	75.6 ± 5.9 (65.4,91.3)	72.9 ± 3.1 (65.4,83.3)	84 ± 4.2 (68.8,91.3)	<0.001
M′L′ (mm)	46.9 ± 4.3 (38.2,66.5)	45.6 ± 3.2 (38.2,56.3)	51.1 ± 4.7 (43.1,66.5)	<0.001
M′L′/ML (%)	62.1 ± 3.9 (53.5,77.4)	62.5 ± 3.6 (53.5,77.4)	60.8 ± 4.5 (53.7,75.6)	<0.01
LD	5.3 ± 1.7 (−1.9,11.7)	5.1 ± 1.6 (1.3,9.9)	5.9 ± 2 (−1.9,11.7)	<0.01
LD/ML (%)	7.0 ± 2.1 (−2.1,13.2)	7.0 ± 2 (1.8,13.2)	7.0 ± 2.2 (−2.1,12.8)	0.93
Double peak type/Single peak type	151/115	119/83	32/33	0.196

SD, standard deviation; ML, mediolateral width of distal femur, M′L′, mediolateral width of anterior resection surface; LD, lateral deviation; LD/ML, ratio between the lateral deviation and mediolateral width.

**Table 5:** Comparison of lateral and medial anterior condylar height according to sex in the previous literature.

Study	Population	Men			Women		
		Lateral	Medial	Ratio (M/L)	Lateral	Medial	Ratio (M/L)
Koh et al. [22] ^1^	Korean	8.3	6.7	0.81	6.7	6.1	0.91
Yang et al. [19] ^2^	Chinese	8.2	3.1	0.38	7.4	3.6	0.49
Poilvache et al. [32] ^1^	Western	13.74	10.63	0.77	12.26	8.96	0.73
Zimmer^®^ [33] ^3^	Western	10.9	6.4	0.59	10.1	5.1	0.50

^1^ Based on TEA. ^2^ Based on sTEA. ^3^ References were not explicitly stated. TEA, transepicondylar axis; sTEA, surgical transepicondylar axis.

## Data Availability

The data presented in this study are available on request from the corresponding author. The data are not publicly available due to privacy.
